# The Role of the Medial Prefrontal Cortex-Amygdala Circuit in Stress Effects on the Extinction of Fear

**DOI:** 10.1155/2007/30873

**Published:** 2007-01-16

**Authors:** Irit Akirav, Mouna Maroun

**Affiliations:** ^1^Department of Psychology, The Brain and Behavior Research Center, University of Haifa, Haifa 31905, Israel; ^2^Department of Neurobiology and Ethology, The Brain and Behavior Research Center, Faculty of Science and Science Education, University of Haifa, Haifa 31905, Israel

## Abstract

Stress exposure, depending on its intensity and duration, affects cognition and learning in an adaptive or maladaptive manner. Studies addressing the effects of stress on cognitive processes have mainly focused on conditioned fear, since it is suggested that fear-motivated learning lies at the root of affective and anxiety disorders. Inhibition of fear-motivated response can be accomplished by experimental extinction of the fearful response to the fear-inducing stimulus. Converging evidence indicates that extinction of fear memory requires plasticity in both the medial prefrontal cortex and the amygdala. These brain areas are also deeply involved in mediating the effects of exposure to stress on memory. Moreover, extensive evidence indicates that gamma-aminobutyric acid (GABA) transmission plays a primary role in the modulation of behavioral sequelae resulting from a stressful experience, and may also partially mediate inhibitory learning during extinction. In this review, we present evidence that exposure to a stressful experience may impair fear extinction and the possible involvement of the GABA system. Impairment of fear extinction learning is particularly important as it may predispose some individuals to the development of posttraumatic stress disorder. We further discuss a possible dysfunction in the medial prefrontal cortex-amygdala circuit following a stressful experience that may explain the impaired extinction caused by exposure to a stressor.

## 1. INTRODUCTION

Pavlovian fear conditioning is an extensively studied model for stress and anxiety-like disorders [1]. In this form of learning, an animal is exposed to pairings of a neutral conditioned stimulus (CS) such as a light or tone, with a fear-inducing unconditioned stimulus (US), such as a mild foot shock, and comes to exhibit a conditioned fear response (CR) to the CS. The CR includes freezing, increased startle reflexes, autonomic changes, analgesia, and behavioral response suppression. Experimental extinction is a behavioral technique leading to suppression of the acquired fear, that is, a decrease in the amplitude and frequency of a CR as a function of nonreinforced CS presentations. Experimental extinction is assumed to reflect an active learning process that is distinct from acquisition of fear and requires additional training to develop [2–5].

While clearly of importance to survival, the expression of emotional associations may become disadvantageous when the conditioned cue ceases to predict the appearance of danger. In that respect, the ability to extinguish emotional responses in the face of a no-longer relevant conditioned cue is an essential part of a healthy emotional memory system, particularly with respect to phobias, panic disorders, and posttraumatic stress disorder [PTSD; [4, 6–8]]. Thus, the suppression of the fear response (i.e., extinction) receives increasing attention, since it could become an effective intervention for the treatment of fear-related disorders.

Extinction suppresses, rather than erases, the original CS-US association. For example, even the completely extinguished fear can be recovered spontaneously after the passage of time [9, 10], or be “reinstated” by presentations of the US alone [11, 12], or be renewed by placing the animal in a context different from the one in which it was extinguished [13]. This is congruent with the notion that extinction is a form of relearning (of a CS-no US or “inhibitory” association) rather than unlearning (of the CS-US association) [14]. Accordingly, one suggestion put forward that extinction suppresses the expression of an intact underlying fear response, and extinction memory is labile and weak compared with the fear conditioning itself. Hence, understanding the factors that facilitate or impair extinction may aid in accelerating behavior therapy for the treatment of anxiety disorders.

Despite the efficacy of behavior therapy for human anxiety disorders, extinction-like treatments require repeated cue exposures and are vulnerable to reversal by a number of environmental factors, particularly stress.

The effects of stressful experiences on cognition are manifested through the activation of multiple mechanisms and operating over different time courses and have been linked to the onset of a variety of affective disorders. Stress can produce deleterious effects on the brain and behavior, and it contributes towards impaired health and an increased susceptibility to disease and mental disorders [15, 16]. Investigations into the interaction between stressful experiences and memory haves focused mainly on the behavioral and neural mechanisms of memory acquisition (i.e., fear conditioning), but not on memory extinction, even though extinction is used for the treatment of psychiatric conditions based on learned fear, such as phobias, panic, generalized anxiety, as well as PTSD.

Extensive evidence indicates that the amygdala and the prefrontal cortex are key structures in the response to stress and its effects on learning and memory. Importantly, it has been shown that extinction of fear memory requires plasticity in both the medial prefrontal cortex (mPFC) and the basolateral amygdala [BLA; [17–19]]. In this review, we will discuss the relevance of the prefrontal cortex-amygdala circuit as a key mechanism for understanding stress-induced alterations occurring during the extinction of fear.

## 2. STRESS AND EXTINCTION

There are intricate relationships between stress and cognitive processes [20]. On the one hand, cognitive processes are necessary to cope adequately with a stressor, both actively and passively, in that a subject has to be aware that there is a stressor and at the same time it has to learn that the stressor can be controlled by an appropriate response. Adaptation to stress occurs when the acquired response is successful in reducing the impact of the stressor. If not, maladaptation may occur. On the other hand, there is strong evidence that stress and stress hormones play an important role in the modulation of cognitive processes. It should be noted that in the fear conditioning paradigm, stress plays a role during conditioning and at least during the first stages of extinction training. Thus, we differentiate here between the aversive situation in the learning paradigm itself, for example, exposure to a foot shock, and the effects of additional exposure to an out-of-context stressor on fear extinction.

**Figure 1:**
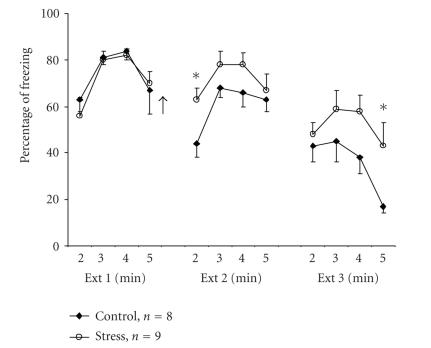
*Stress impairs extinction of contextual fear conditioning.* Rats were given 3 mild foot shocks in the conditioning chamber. On the next day, the rats were placed in the extinction (Ext) chamber for 5 minutes and no shock was administered (Ext 1; the last 4 minutes are presented since all animals showed high levels of freezing in the first minute). Immediately afterwards, the animals were returned to their home cage (control) or placed on an elevated platform for 30 minutes (stress). Animals were exposed to additional 5 minutes in the extinction chamber, without shocks, on days 3 (Ext 2) and 4 (Ext 3). The stressed animals showed significantly higher levels of freezing compared with the control group during the second minute of Ext 2 (∗; *P* < .05) and the fifth minute of Ext 3 (∗; *P* < .05). Arrow denotes time of exposure to stress.

When examining the effects of exposure to an out-of-context stressor on fear extinction, we found that the stressor increased resistance to extinction (H. Reizel, I. Akirav, and M. Maroun, unpublished observation; Figure 1). Specifically, after contextual fear conditioning (using a US of 3 foot shocks of 0.5 mA each), control rats gradually extinguished their freezing (CR) when placed in the extinction box (CS) for 3 consecutive days, for 5 minutes each time. By contrast, the experimental rats were exposed to the out-of-context stressor on being placed on an elevated platform for 30 minutes immediately after the first extinction session. Animals placed on the platform exhibited behavioral “freezing,” that is, immobility for up to 10 minutes, defecation, and urination [21, 22]. This stressor was found to increase plasma corticosterone levels by 38% as compared with naïve rats [23] and we have recently found that it impairs long-term potentiation in the CA1 area of the hippocampus and in the BLA-medial prefrontal pathway [24]. In the contextual fear extinction experiment, the stressed rats showed increased levels of freezing in the extinction box even 48 hours after a single exposure to the elevated platform. This suggests that exposure to the stressor had the long-term effect of impairing the extinction of fear.

We found that exposure to stress had a similar effect on consolidation of the extinction of auditory fear conditioning (see later, see below, or ahead). The impairing effects of the elevated platform on auditory fear extinction also persisted for 48 hours following exposure to the stressor. Consistent with our results, Izquierdo et al. [25] reported that exposure to three episodes of stress ending 24 hours before fear conditioning significantly attenuated the rate of cued fear extinction relative to nonstressed controls. Shumake et al. [26] showed that rats that were selectively bred for increased susceptibility to learned helplessness show resistance to extinction of conditioned fear. Furthermore, Kellett and Kokkinidis [27] showed that amygdala kindling, which enhances emotionality, impaired the extinction of fear-potentiated startle, and rats showed increased levels of fear. They also found that electrical stimulation of the amygdala restored extinguished fear responses and that the fear reinstatement was specific to the extinction context. In a study with rainbow trout, Moreira et al. [28] compared two lines of fish that exhibit divergent endocrine responsiveness to stressors: the high-responders (HR) and low-responders (LR; the “stressed”). Postconditioning, the fish were tested by presentation of the CS at weekly intervals for 4 weeks, with no further reinforcement, and the extinction of the CR in the two lines was compared. The number of individuals within each line whose plasma cortisol levels indicated a stress response when exposed to the CS was significantly greater among the LR than HR fish at 14 and 21 days, with no HR fish falling into the stress-response category at 21 days. Thus, the stressed fish did not extinguish as well as the HR fish.

It is important to understand why exposure to stress impairs extinction learning, and here we put forward four possible explanations. One possibility is that extinction memory is labile and weak compared with fear conditioning itself, and thus exposure to a stressful experience interferes with the process of extinction learning or with the retrieval of information. Second, it has been shown that a stressful experience following or preceding a threatening or fear-related learning event enhances retention [29]. However, in extinction, the animals need to learn to suppress their fear response that is associated with the CS. Thus, the aversiveness of the stressful experience may counteract the extinguished emotional response. Further, it is possible that preexposure to the stressful experience increases resistance to extinction through sensitization, leading to the occurrence of a conditioned fear response even to a less intense “reminder” of the original US. Thus, retrieval of the CS-US association (i.e., acquisition) overcomes the CS-no US association (i.e., extinction) following the sensitization effect, making extinction more difficult to learn. However, this can hardly explain why exposure to an unrelated stressful experience, such as an elevated platform, should sensitize the animals to respond as if to the US during extinction training. A fourth possibility is that resistance to extinction is not related to sensitization or to the enhancement of an unspecific fear response. Accordingly, if the enhanced fear memory is expressed only when stressed animals are exposed to the CS, it may indicate that this response is sustained by associative learning, and thus the increased freezing behavior of stressed animals could be attributable to an attenuation of the extinction process, rather than to enhanced fear acquisition, although the latter remains a possibility [4].

It is usually assumed that stressful life events interfere with our ability to acquire new information. Yet, previous exposure to both acute and chronic stressful events can positively affect classical conditioning tasks, including fear conditioning [29–33]. Reports to date regarding the effects of stress on fear extinction show that exposure to stress increases resistance to extinction, that is, it impairs extinction acquisition and consolidation, which reduces the extent to which extinction is able to offset a fear response. In contrast, studies addressing the relationship between stress and the acquisition of new fear memories show that exposure to a stressful experience facilitates fear learning, so further enhancing the fear response. For example, previous exposure to a restraint session increased fear conditioning in a contextual fear paradigm [33]. Similarly, Rau et al. [34] have shown that preexposure to a stressor of repeated foot shocks enhanced conditional fear responses to a single context-shock pairing. Cordero et al. [29] have shown that a single exposure to an aversive stimulus is sufficient to facilitate context-dependent fear conditioning, and suggested increased glucocorticoid release at training in the mechanisms mediating the memory-facilitating effects induced by prior stressful experiences. These studies corroborate others showing that if an animal learns a stressful task, then the consolidation of this task may be enhanced by stress and that its end product, corticosterone, may be secreted during the task [35–37]. This was found to be the case in a variety of emotionally arousing tasks, such as inhibitory avoidance, spatial learning, discrimination learning, and fear conditioning [38–44].

## 3. THE NEURAL BASIS OF FEAR EXTINCTION

The basolateral amygdala (BLA) plays a pivotal role in the consolidation of memories related to fear and emotions, and in the initiation of responses to stressful events [37, 45–50]. Moreover, the BLA is significantly involved in both the formation and extinction of fear memory [17, 51–54]. For example, microinfusions of a protein synthesis inhibitor to the amygdala prevented recall of extinction after 30 minutes, and infusion of N-methyl-D-aspartate (NMDA) receptor antagonists or mitogen-activated protein kinase inhibitors to the BLA prevented across-day extinction of fear-potentiated startle [17, 54–56]. In another study [57], BLA lesions severely attenuated expression of previously acquired fear memory. Also, infusion of an NMDA agonist into the amygdala facilitated fear extinction [58, 59].

Another brain structure that is known to play an important role, not only in the regulation of emotion, but also in the integration of affective states with appropriate modulation of autonomic and neuroendocrine stress regulatory systems [60], is the medial prefrontal cortex (mPFC). The mPFC provides an interface between limbic and cortical structures [61] and regulates the stress-induced activity of the hypothalamus-pituitary-adrenal (HPA) axis [62, 63].

The mPFC is important in long-term fear extinction memory. Specifically, lesions or inhibition of protein synthesis in the infralimbic part of the medial PFC impair recall of extinction of conditioned fear [18, 19, 64, 65]. Furthermore, mPFC stimulation that mimics extinction-induced tone responses reduces conditioned fear [66, 67], and stimulating the mediodorsal thalamic inputs to the mPFC is associated with extinction maintenance [68, 69]. Moreover, functional imaging studies in human subjects indicate that the mPFC is engaged during extinction [70] and that subjects with PTSD have reduced mPFC activity during trauma recall [71]. Furthermore, Miracle et al. [72] have shown that one week of restrained stress had the effect of impairing recall of extinction of conditioned fear, and suggested that this is due to deficits in the mPFC caused by exposure to stress. Recently, it has been reported that stress exposure that impairs fear extinction also caused retraction of terminal branches of apical dendrites of infralimbic neurons [25].

## 4. THE ROLE OF GABA IN EXTINCTION OF FEAR

In addition to evidence indicating that extinction of fear memory requires plasticity in both the mPFC and the BLA [17–19], recent studies further point to a dysfunctional interaction between the prefrontal cortex and the amygdala in the failure to extinguish conditioned fear. These studies indicate that the mPFC has a function in the inhibition of emotions through its projections to the amygdala [73] and are in line with Pavlov's [74] view that extinction learning involves inhibitory cortical circuits that reduce the CS-evoked conditioned response.

The glutamatergic efferents from the mPFC synapse on amygdala gamma-aminobutyric acid (GABA)ergic neurons [75], and through this, may provide important inhibitory input to the amygdala. Of particular interest is the projection from the infralimbic region of the PFC (which, together with the prelimbic cortex, comprises the ventromedial PFC) to the capsular division of the central nucleus of the amygdala [76]. The capsular division of the central nucleus contains GABA-ergic intercalated cells that have been shown to exert powerful inhibitory control over central nucleus neurons that project out of the amygdala [77–79]. Infralimbic input to intercalated cells could be a pathway by which infralimbic tone responses inhibit the expression of conditioned fear (e.g., reduce freezing) [80].

The anatomical data described for the interaction between these two structures pinpoint the crucial role the neurotransmission of GABA may play in the extinction of fear. Indeed, a substantial number of studies have demonstrated that the BLA contains a powerful inhibitory circuit that uses GABA as a neurotransmitter [81–83]. Moreover, the BLA has larger amounts of benzodiazepine/GABA_A_ receptors than any other amygdala nucleus [84], explaining why the infusion of benzodiazepines or GABA_A_ agonists into the BLA reduces fear conditioning and anxiety [85–88]. Coincidently, local blockade of these receptors attenuates the anxiolytic influence of systemic benzodiazepines [89]. Recently, Rodríguez Manzanares et al. [33] have shown that stress attenuates inhibitory GABA-ergic control in the BLA, leading to neuronal hyperexcitability and increased plasticity that facilitates fear learning. Based on these data, it can be concluded that GABA-ergic mechanisms in the amygdala play a major role in controlling the emotional consequences of stress, and may thus affect extinction of fear.

Benzodiazepines have long been used to treat anxiety and are particularly appropriate in short-term treatment situations [8]. Direct modulation of GABA-ergic neurons, through the benzodiazepine-binding site, down regulates memory storage processes and specifically affects learned fear responses. On the other hand, benzodiazepine release could be modulated by the anxiety and/or stress associated with different types of learning [90].

Much research is directed at exploring the involvement of GABA in inhibiting learned fear responses. Although several studies support the central role GABA neurotransmission plays in extinction, there are different reports regarding whether this role is to facilitate or impair extinction [26, 91–95]. Using direct modulation of GABA-ergic neurons, it has been shown that the benzodiazepine inverse agonist FG7142, which attenuates the effect of GABA at its receptor, retards extinction of conditioned fear [91, 96]. Likewise, McCabe et al. [97] have shown that benzodiazepine agonists administered to mice following training significantly facilitated extinction during a food-reinforced lever-press procedure. Potentiation of GABA by the benzodiazepine agonist chlordiazepoxide administered prior to extinction sessions facilitated extinction in a paradigm of operant responding for food reinforcement [98]. By contrast, systemic administration of the GABA_A_ antagonist picrotoxin, after the extinction of inhibitory avoidance learning, enhanced extinction retention during testing [93], and the GABA_A_-positive allosteric modulator diazepam impaired extinction retention when administered before extinction in a shuttle avoidance task [95].

There are also a number of ways of modulating GABA-ergic functions indirectly. For example, cannabinoid (CB1) receptors and gastrin-releasing peptide receptors are both located on GABA-containing interneurons. Endogenous cannabinoids, acting at the CB1 receptor, facilitated the extinction of aversive memories [92], and blocking the action of gastrin-releasing peptide, by genetically removing its receptor, retards extinction of learned fear responses [26]. Recently, Azad et al. [99] have shown that CB1 receptors reduce GABA-ergic synaptic transmission in the amygdale, and consequently facilitate extinction of aversive memories. Chhatwal et al. [100] showed that gephyrin mRNA and protein levels in the BLA significantly increased after fear extinction training, suggesting that the modulation of gephyrin and GABA_A_ receptor expression in the BLA may play a role in the experience-dependent plasticity underlying extinction.

**Figure 2:**
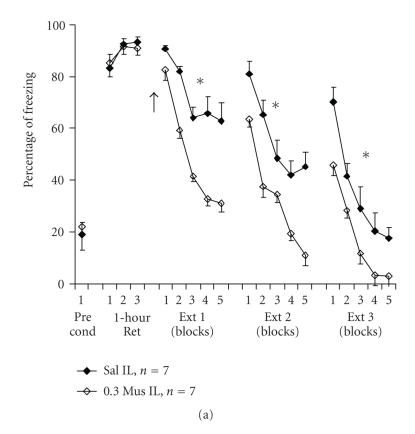

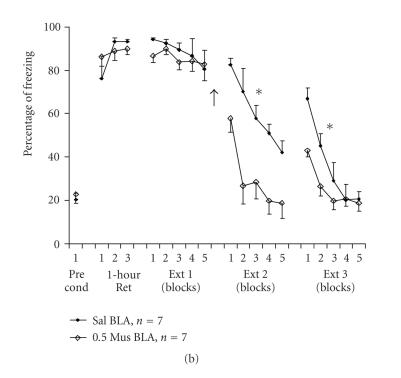
(a) *A low volume of muscimol microinfused into the infralimbic cortex before extinction training facilitates extinction learning.* Rats received 7 pairings of a tone with a foot shock in the conditioning chamber. After 1 hour, three tones were delivered in the absence of foot shock (1-hour Ret). On the next day, the animals were microinfused with a total of 0.3 *μ*l saline (Sal) or muscimol (0.3 Mus) to the infralimbic cortex (IL) and were exposed to 15 tones without foot shocks (Ext 1; presented as 5 blocks of 3 trials). Animals were exposed to additional 15 tones on days 4 (Ext 2) and 5 (Ext 3), without further administration of the drug. Muscimol IL animals showed significantly lower levels of freezing compared with the saline group in Ext 1 (∗; *P* < .001), Ext 2 (∗; *P* < .01) and Ext 3 (∗; *P* < .05). This supports a selective involvement of the IL in facilitating extinction of conditioned fear (see Akirav et al. [51]). Arrow denotes time of drug infusion. The Pre cond data points indicate the amount of freezing exhibited by rats prior to commencement of fear conditioning. (b) *A low volume of muscimol microinfused to the basolateral amygdala following a short extinction training session facilitates extinction consolidation.* Rats received 7 pairings of a tone with a foot shock in the conditioning chamber. After 1 hour, three tones were delivered in the absence of foot shock (1-hour Ret). On the next day, the animals underwent a short extinction training session consisting of 5 tones (Ext 1; presented as 5 trials), and were thereafter microinfused with a total volume of 0.5 *μ*l saline (Sal) or muscimol (0.5 Mus) to the basolateral amygdala (BLA). On days 4 and 5 (Ext 2 and Ext 3, resp.), the animals were exposed to 15 tones without foot shocks (presented as 5 blocks of 3 trials). The BLA muscimol group showed significantly reduced levels of freezing compared with the other two groups during Ext 2 (∗; *P* < .001) and Ext 3 (∗; *P* < .05). This supports the selective involvement of the BLA in facilitating consolidation of extinction of conditioned fear (see Akirav et al. [51]). Arrow denotes time of drug infusion. The Pre cond data points indicate the amount of freezing exhibited by rats prior to commencement of fear conditioning.

Using a low dose of the GABA_A_ agonist muscimol, we recently found [51] that muscimol infused to the infralimbic area before extinction training (see Figure 2(a)) resulted in long-term facilitation of extinction. By contrast, where infusion of muscimol to the infralimbic area followed extinction training, no such effect was observed, regardless of the length of the extinction training period (5 or 15 trials; data not shown). However, infusion of muscimol to the BLA following a short (5-trial) extinction session facilitated extinction for at least 48 hours post-drug-infusion (see Figure 2(b)). The differences between the temporal parameters of the effects of muscimol in the infralimbic cortex compared to the BLA suggest differential involvement of these structures in long-term extinction of fear memory. We propose that GABA_A_ neurotransmission in the infralimbic cortex plays a facilitatory role in triggering the onset of fear extinction and its maintenance, whereas in the BLA, GABA_A_ neurotransmission facilitates extinction consolidation.

Overall, the data suggest that manipulation of GABA transmission may have very different effects depending on whether it is administered pre- or postextinction training or before a retention test, and depending also on the behavioral paradigm used. Future studies are required to understand these discrepancies.

**Figure 3:**
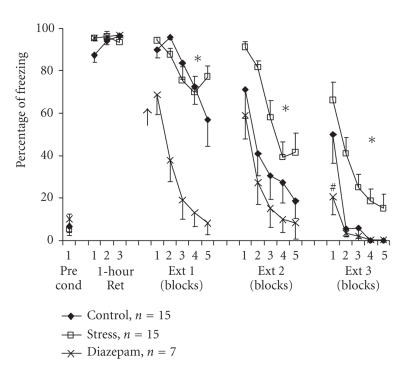
*Diazepam overcomes stress-induced impairment of the extinction of auditory fear.* Rats were exposed to 3 pairings of a tone with a mild foot shock in the conditioning chamber. On the next day, control animals remained in their home cages, “diazepam” group animals were injected with diazepam (2 mg/kg, IP) 20 minutes before being placed on an elevated platform for 30 minutes, while “stress” group animals were placed directly onto the elevated platform for 30 minutes, without prior administration of the drug. Immediately afterwards, animals were taken for extinction training and were exposed to15 tones (Ext 1) with no shock. Animals were exposed to an additional 15 tones on days 3 (Ext 2) and 4 (Ext 3) with no drug or shock. There were significant differences between the diazepam group and the other groups during Ext 1 (*P* < .001). On Ext 2 and Ext 3, the stress group was significantly different from the control (Ext 2: *P* < .05, Ext 3: *P* < .01) and the diazepam (Ext 2: *P* < .01, Ext 3: *P* < .001) groups. Arrow denotes time of drug infusion. The Pre cond data points indicate the amount of freezing exhibited by rats prior to commencement of fear conditioning.

While examining the involvement of GABA in the effects of stress on fear extinction, we found that systemic administration of the benzodiazepine agonist diazepam reversed the resistance to extinction induced by exposure to an out-of-context stressor (see Figure 3). After classical auditory fear conditioning (3 CS-US pairings of a tone with a foot shock of 0.5 mA), control rats that were exposed to the tone without shock gradually extinguished their freezing (CR) in response to the tone during extinction training. At the end of the third extinction session, their freezing levels dropped to zero. Rats that were exposed to an out-of-context stressor (i.e., animals that were placed on an elevated platform for 30 minutes) before the first extinction training session showed increased levels of freezing in response to the tone even 48 hours after the stressor (i.e., showed resistance to extinction). A single injection of diazepam (2 mg/kg, IP) 20 minutes before exposure to the out-of-context stressor significantly facilitated extinction compared with the control and stress groups as manifested by reduced freezing levels in the first extinction session. On the second and third sessions of extinction training, the response of the diazepam-stress group was no different to that of the control group, with the former group also exhibiting significantly less freezing than the stressed rats that had not first received diazepam. Hence, treatment with diazepam reversed the impairing effect of exposure to stress on fear extinction. Further experiments to elucidate the possible role GABA plays in the BLA and the mPFC in preventing stress-associated impairments of extinction are required.

A problem associated with the use of anxiolytic and anxiogenic compounds in studies of extinction, however, is the possibility of state dependency as opposed to a true effect on the suppression of the learning process [101]. That is, it is possible that a drug administered before or immediately following extinction produces an internal state, or drug context, that is discriminable to the animal [102]. However, in our experiment, the effect was probably not due to state dependence because the stressed animals that were treated with diazepam showed less freezing (i.e., more extinction) than the stressed animals that were treated with saline, even 24 and 48 hours after a single injection.

To conclude, the present results demonstrate that pretreatment with the benzodiazepine tranquilizer diazepam reverses the CR-enhancing effects of the elevated platform experience. These findings suggest that benzodiazepines may prevent the augmentation of the trauma-related symptoms seen in phobia and PTSD patients that are caused by exposure to a stressful experience.

## 5. EXTINCTION OF FEAR: INTERPLAY FOR DOMINANCE BETWEEN THE AMYGDALA AND THE PREFRONTAL CORTEX

Recent observations provide direct physiological support that the mPFC reduces fear responses by reducing amygdala output [66, 103, 104]. For example, Milad and Quirk [66] found that stimulation of the mPFC decreases the responsiveness of central amygdala neurons that regularly fire in response to the CS only when animals are recalling extinction of a fear task learned using that CS. Additionally, Morgan et al. [64] reported that rats with mPFC lesions had an increased resistance to extinction. They proposed that connections between the mPFC and amygdala normally allow the organism to adjust its emotional behavior when environmental circumstances change, and that some alteration in this circuitry, causing a loss of prefrontal control of the amygdala, might underlie the inability of persons with anxiety disorders to regulate their emotions.

If the mPFC normally inhibits the amygdala as an active component of extinction of fear conditioning, then when the mPFC is inhibited or suppressed, emotional associations mediated by the amygdala may be not inhibited during nonreinforcement. As a result, conditioned responding may be prolonged over time [64].

A combination of changes throughout this circuit is important in generating stress-induced changes in emotionality. The mPFC may have a regulatory role in stress-induced fear and anxiety-like behaviors through inhibitory effects on amygdala output and processing [105]. Indeed, extensive evidence supports the notion that the BLA is a site of plasticity for fear conditioning [104, 106], and that the BLA is extensively connected with the central nucleus of the amygdala [107, 108]. In turn, the central nucleus projects to the paraventricular nucleus of the hypothalamus [109], thereby providing the most likely route for any BLA-dependent effects on stress-induced HPA output.

We would like to take this a step further, and suggest a possible mode of action for the mPFC-amygdala circuit in fear extinction under stressful conditions. Accordingly, under normal conditions of fear suppression, the mPFC is activated and inhibits amygdala output. This dominance of the mPFC results in normal suppression of fear, and in consequence promotes extinction of fear. However, exposure to a stressful experience may reduce medial PFC inhibition of the amygdala, and as a result the amygdala takes control to assure defensive behaviors and becomes dominant. The expected consequence is interference in the suppression of the fear response, that is, impaired extinction learning. Therefore, exposure to a stressful experience would result in reduced mPFC activity leading to resistance to extinction and inappropriate and exaggerated fear responses, as seen in PTSD patients. Indeed, abnormally low PFC activity together with abnormally high amygdala activity were found in PTSD patients, when reexposed to traumatic reminders [110]. Accordingly, deficits in extinction of conditioned fear as a result of exposure to a stressful experience are proposed to contribute to the sustained anxiety responses seen in PTSD.

**Figure 4:**
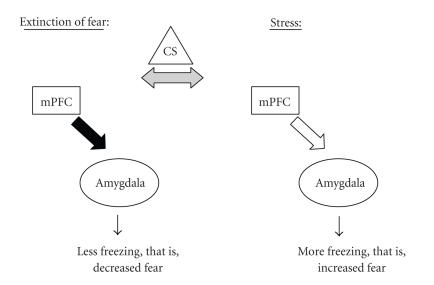
*A possible mode of action for the medial prefrontal cortex-amygdala circuit in fear extinction under normal and stressful conditions.* Under normal conditions of fear suppression, the medial prefrontal cortex (mPFC) is activated and inhibits amygdala output (filled arrow). This dominance of the mPFC results in less freezing in response to a conditioned stimulus (CS; i.e., extinction). However, under stressful conditions, the inhibitory action of the mPFC on the amygdala is reduced (empty arrow), the amygdala dominates (indicated by the bold circle around the amygdala) and the result is more freezing in response to a CS (i.e., impaired extinction).

Figure 4 schematically summarizes this idea and shows that during extinction of fear, the mPFC is activated and acts to inhibit the amygdala in order to reduce fear, resulting in less freezing (i.e., extinction). However, exposure to stress at a critical time with respect to extinction learning activates the amygdala to increase fear and the result is more freezing (i.e., resistance to extinction). Therefore, according to our proposed model, the stressor shifts the dominance from the mPFC to the amygdala and, as a consequence, extinction of fear is impaired.

Our model is consistent with the data shown in Figure 1, which demonstrate that exposure to a stressful experience results in resistance to extinction in the stressed group compared with the nonstressed group. Whether this effect is due to a reduction in mPFC modulation of amygdala output, and to the involvement of GABA-based mechanisms acting on the PFC-amygdala circuit, still needs to be examined. Our model is also consistent with the suggestion put forward by Quirk and Gehlert [111] that deficient inhibitory tone in the amygdala due to decreased inhibition from the prefrontal cortex could lead to overexpression of conditioned responses, producing pathological states such as anxiety disorders and drug-seeking behavior.

## 6. PERSPECTIVES

Pathological fear and anxiety, such as that exhibited by PTSD sufferers, may be the manifestation of abnormal modulations in the activity of the amygdala and the mPFC, and in their interaction. PTSD is defined as symptoms of reexperiencing the trauma, avoidance of associated stimuli and hyperarousal symptoms, suggesting a heightened fear response, and it has been proposed that PTSD symptoms reflect amygdala hyperresponsivity to fear-related stimuli, with a concomitant lack of “top-down” prefrontal inhibition. This proposal is supported by neuroimaging studies of PTSD patients, which observed abnormal reductions in mPFC activity [71, 112, 113], as well as enhanced and distinctive amygdala engagement [114, 115], particularly for combat PTSD veterans [113]. In line with this, fMRI and PET data have shown significant inverse correlations between the functional activity of the mPFC and the amygdala [116, 117]. Collectively, these data provide strong support for the hypothesis that PTSD is characterized by a failure of the mPFC to sufficiently inhibit the amygdala.

There is clinical interest in the effects of stress on fear extinction learning as a model for the mechanisms operating in PTSD, as well as interest in means to improve therapeutic outcomes following fear-extinction-based strategies. Future therapies aimed at increasing the inhibitory tone in the amygdala, either locally or via the prefrontal cortex, may accelerate extinction and may help in the treatment of anxiety disorders.
